# Crystal structure and Hirshfeld surface analysis of bis­{(*Z*)-*N*′-[(*E*)-(furan-2-yl)methyl­idene]carbamo­hydrazono­thio­ato}nickel(II) methanol disolvate

**DOI:** 10.1107/S2056989023005182

**Published:** 2023-06-30

**Authors:** Asmet N. Azizova, Gunay Z. Mammadova, Sevim Türktekin Çelikesir, Mehmet Akkurt, Ajaya Bhattarai

**Affiliations:** a Department of Synthesis of Biologically Active Compounds, Scientific Research Center, Azerbaijan Medical University, Samed Vurgun St. 167, Az 1022 Baku, Azerbaijan; b Organic Chemistry Department, Baku State University, Z. Xalilov Str. 23, Az 1148 Baku, Azerbaijan; c Department of Physics, Faculty of Sciences, Erciyes University, 38039 Kayseri, Türkiye; d Department of Chemistry, M.M.A.M.C (Tribhuvan University), Biratnagar, Nepal; Texas A & M University, USA

**Keywords:** crystal structure, ligands, distorted square-planar geometry, hydrogen bonds, Hirshfeld surface analysis

## Abstract

In the title complex, [Ni(C_6_H_6_N_3_OS)_2_]·2CH_3_OH, the Ni^II^ atom is coordinated by the S and N atoms of two *N*′-[(*Z*)-(furan-2-yl)methyl­idene]carbamohydrazono­thioic acid ligands in a distorted square-planar geometry. The two mutual ligands bound to Ni^II^ are also connected by C—H⋯S inter­actions, while the H atoms of the NH_2_ group of the ligands form *R* ^4^_4_(8) motifs with the O atoms of the solvent ethyl alcohol mol­ecules. At the same time, the OH groups of the solvent ethyl alcohol mol­ecules form parallel layers to the (011) plane by the O—H⋯N inter­actions with the ligand N atom that is not bonded to the Ni^II^ atom.. The layers are connected by van der Waals inter­actions. A Hirshfeld surface analysis indicates that the most important contacts are H⋯H (37.7%), C⋯H/H⋯C (14.6%), O⋯H/H⋯O (11.5%) and S⋯H/H⋯S (10.6%).

## Abstract 2

In the title complex, the Ni^II^ atom is coordinated by the S and N atoms of two *N*′-[(*Z*)-(furan-2-yl)methyl­idene]carbamohydrazono­thioic acid ligands in a distorted square-planar geometry.

## 1. Chemical context

Hydrazones have been used extensively as substrates in organic synthesis (Polyanskii *et al.*, 2019 ▸; Shikhaliyev *et al.*, 2019 ▸; Safavora *et al.*, 2019 ▸; Zubkov *et al.*, 2018 ▸) and multidentate ligands (Gurbanov *et al.*, 2020*a* ▸,*b* ▸; Gurbanov *et al.*, 2022 ▸) while their complexes have been found to possess a wide variety of useful properties. Thus, they can be used as sensor or analytical reagents, catalysts and building blocks in crystal engineering (Ma *et al.*, 2021 ▸; Mahmudov *et al.*, 2010 ▸; Mahmoudi *et al.*, 2017*a* ▸,*b* ▸). Not only because of their coordination ability, but also the attached substituents, the inter­molecular non-covalent inter­actions direct the functional properties as well as the supra­molecular chemistry of hydrazones (Abdelhamid *et al.*, 2011 ▸; Khalilov *et al.*, 2021 ▸; Kopylovich *et al.*, 2011 ▸; Mahmudov *et al.*, 2015 ▸;). In fact, hydrogen and chalcogen bonds and other types of weak inter­actions have been well employed in the decoration of the secondary coordination sphere of transition-metal complexes (Mahmoudi *et al.*, 2019 ▸; Mahmudov *et al.*, 2012 ▸, 2022 ▸). We have synthesized a new Ni^II^ complex of a (*E*)-2-(furan-2-yl­methyl­ene)hydrazine-1-carbo­thio­amide ligand and studied its crystal structure.

## 2. Structural commentary

**Figure 1:**
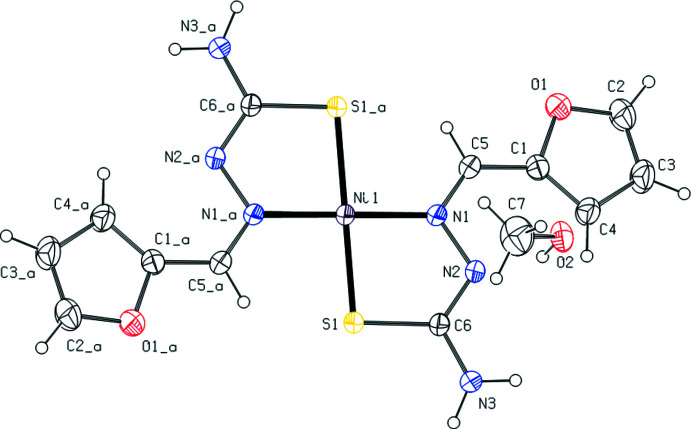
The mol­ecular structure of the title compound, with atom labelling. The displacement ellipsoids are drawn at the 30% probability level.

Fig. 1 ▸ shows the arrangement of the complex mol­ecules in the unit cell. The Ni^II^ atom is coordinated by the S and N atoms of two *N*′-[(*Z*)-(furan-2-yl)methyl­idene]carbamohydrazono­thioic acid ligands in a distorted square-planar geometry. The ligands assume a *trans* arrangement with respect to each other around the Ni^II^ ion, which lies on a crystallographic inversion centre at (−*x* + 1, −*y*, −*z* + 1). The Ni—S [2.1818 (6) Å] and Ni—N [1.9055 (17) Å] bond lengths lie within the range of those found in related structures. 
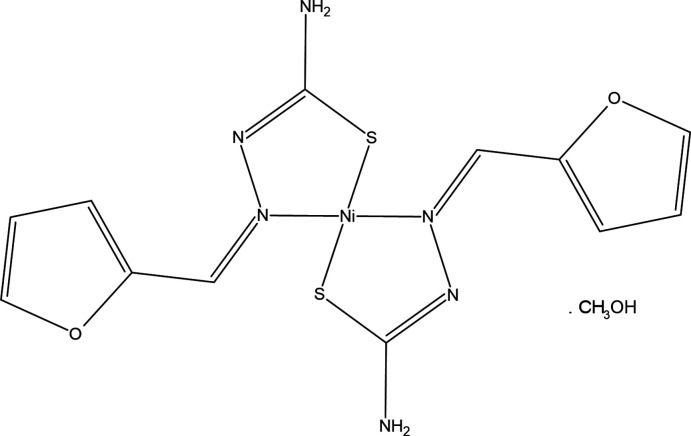


## 3. Supra­molecular features and Hirshfeld surface analysis

**Figure 2:**
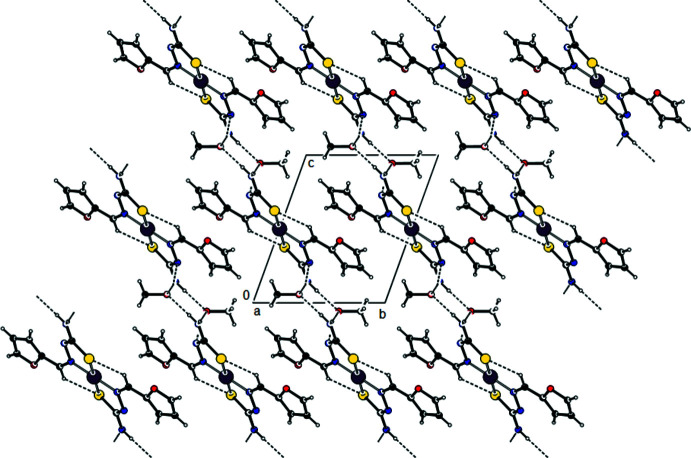
A view along the *a* axis of the crystal packing of the title compound. The O—H⋯N, N—H⋯O and C—H⋯S hydrogen bonds are shown as dashed lines.

In the crystal, the two mutual ligands bound to Ni^II^ are also linked by C—H⋯S inter­actions, while the H atoms of the NH_2_ group of the ligands form 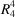 (8) motifs (Bernstein *et al.*, 1995 ▸; Tables 1 ▸ and 2 ▸; Fig. 2 ▸) with the O atoms of the solvent ethyl alcohol mol­ecules. At the same time, the OH groups of the solvent ethyl alcohol mol­ecules form parallel layers to the (011) plane by the O—H⋯N inter­actions with the ligand N atom that is not bonded to the Ni^II^ atom (Figs. 2 ▸, 3 ▸ and 4 ▸). These layers are connected by van der Waals inter­actions.

**Figure 5:**
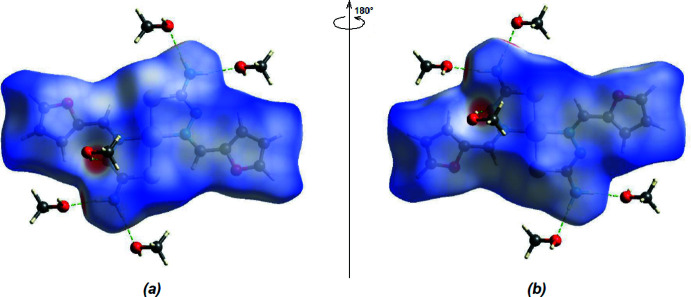
(*a*) Front and (*b*) back sides of the three-dimensional Hirshfeld surface of the title compound mapped over *d*_norm_.

A Hirshfeld surface analysis was carried out using *CrystalExplorer 17.5* (Spackman *et al.*, 2021 ▸) to analyse the inter­molecular inter­actions. The three-dimensional Hirshfeld surface mapped over the normalized contact distance (*d*_norm_) is shown in Fig. 5 ▸. The bright-red spots indicate shortened contacts, and correspond to the O—H⋯N and N—H⋯O inter­molecular hydrogen bonds.

**Figure 6:**
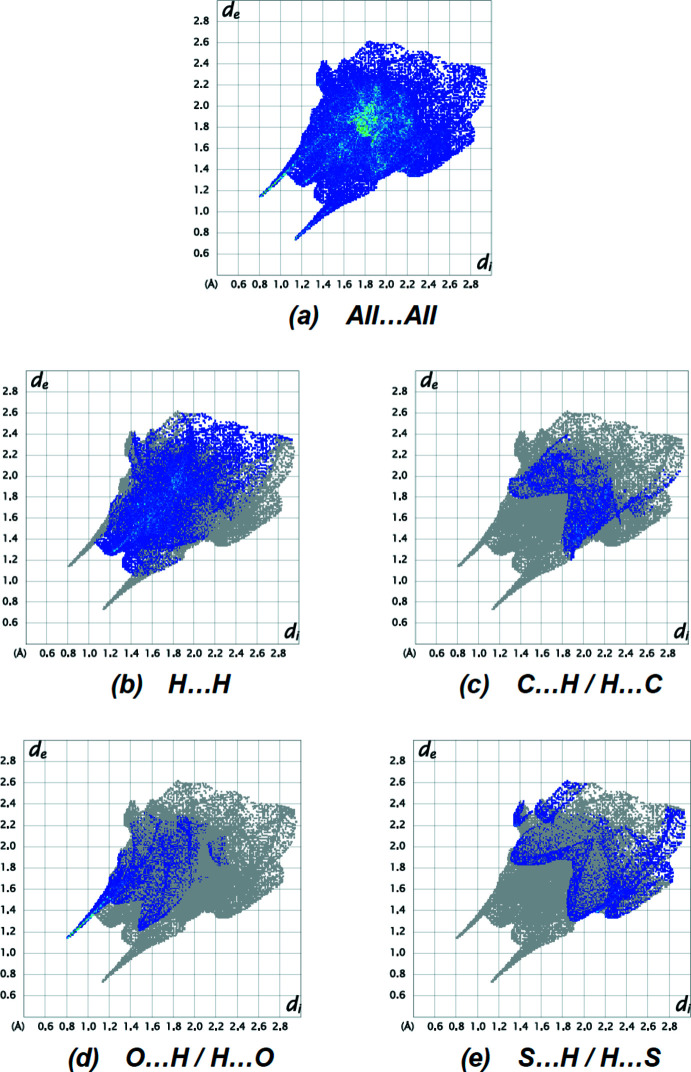
The two-dimensional fingerprint plots of the title compound, showing (*a*) all inter­actions, and delineated into (*b*) H⋯H, (*c*) C⋯H/H⋯C, (*d*) O⋯H/H⋯O and (*e*) S⋯H/H⋯S inter­actions. [*d*_e_ and *d*_i_ represent the distances from a point on the Hirshfeld surface to the nearest atoms outside (external) and inside (inter­nal) the surface, respectively].

The two-dimensional fingerprint plots show the H⋯H (Fig. 6 ▸ *b*; 37.7%) contacts to be the most common, followed by C⋯H/H⋯C (Fig. 6 ▸ *c*; 14.6%), O⋯H/H⋯O (Fig. 6 ▸ *d*; 11.5%) and S⋯H/H⋯S (Fig. 6 ▸ *e*; 10.6%) contacts. The N⋯H/H⋯N (8.5%), O⋯C/C⋯O (4.9%), Ni⋯H/H⋯Ni (3.2%), O⋯N/N⋯O (2.2%), N⋯C/C⋯N (1.9%), C⋯C (1.8%), S⋯C/C⋯S (1.1%), S⋯S (0.7%), O⋯O (0.7%),S⋯O/O⋯S (0.5%) and Ni⋯C/C⋯Ni (0.2%) contacts have little directional influence on the mol­ecular packing.

## 4. Database survey

A search of the Cambridge Structural Database (ConQUEST version 2022 3.0; Groom *et al.*, 2016 ▸) for one of the Ni atoms plus ligands in the title compound yielded 14 structures that have the same framework as the title compound. FUTRAN (Puranik *et al.*, 1987 ▸) appears to be the same structure, without any solvent, and NOQCUS (Rodríguez-Argüelles *et al.*, 2009 ▸) is the same with a dimethyl sulfoxide solvent mol­ecule; the other 12 have alkyl or phenyl groups attached.

In the crystal of FUTRAN, Ni ^II^ is in the distorted square planar ligand field of the N_2_S_2_ chromophore. The thio­semicarbazonato group is planar with Ni—S = 2.149 (1) Å and Ni—N(2) = 1.921 (2) Å. The coordination around Ni is *trans* planar with respect to the two S and two N atoms. The furan ring plane is at an angle of 3(1)° to the coordination plane. In the crystal of NOQCUS, the coordination environment around the nickel(II) ion is totally planar, as the NiN_2_S_2_ chromophore lies on its least-squares calculated plane and the four angles formed by the metal centre with the four donor atoms add up to exactly 360°. The Ni—N and Ni—S distances are within the usual range. This plane forms a 18° angle with the uncoordinated furan ring, which is also highly planar.

## 5. Synthesis and crystallization

17 mg (0.1 mmol) of (*E*)-2-(furan-2-yl­methyl­ene)hydrazine-1-carbo­thio­amide were dissolved in 30 mL of methanol then 13 mg (0.05 mmol) of *Ni*(OOCCH_3_)_2_·4H_2_O were added. The reaction mixture was kept in air at room temperature for slow evaporation. After *ca* 2–3 d, orange crystals, suitable for X-ray analysis, were formed.

Yield 81%, soluble in DMSO, ethanol and di­methyl­formamide and insoluble in non-polar solvents. Elemental analysis: C_14_H_20_N_6_NiO_4_S_2_ (*M = 459*.17); C 36.61 (calc. 36.62); H 4.35 (4.39); N 18.26 (18.30) %. IR (KBr): 3372 ν(OH), 2965 and 2854 ν(NH), 1643 ν(C=N) cm^−1^.

## 6. Refinement

Crystal data, data collection and structure refinement details are summarized in Table 3 ▸. C-bound H atoms were positioned geometrically (C—H = 0.93 and 0.96 Å) and refined using a riding model with *U*_iso_(H) = 1.2 or 1.5*U*_eq_(C). O- and N-bound H atoms were located in difference Fourier maps [O2—H2*O* = 0.90 Å, N3—H3*A* = 0.90 Å, N3—H3*B* = 0.90 Å] and refined with *U*_iso_(H) = 1.2*U*_eq_(N) and 1.5*U*_eq_(O), with their positions fixed. Two reflections (001) and (010), affected by the beam stop, were omitted in the final cycles of refinement.

## Supplementary Material

Crystal structure: contains datablock(s) I, global. DOI: 10.1107/S2056989023005182/jy2031sup1.cif

Structure factors: contains datablock(s) I. DOI: 10.1107/S2056989023005182/jy2031Isup2.hkl

CCDC reference: 2269284

Additional supporting information:  crystallographic information; 3D view; checkCIF report

## Figures and Tables

**Figure 3:**
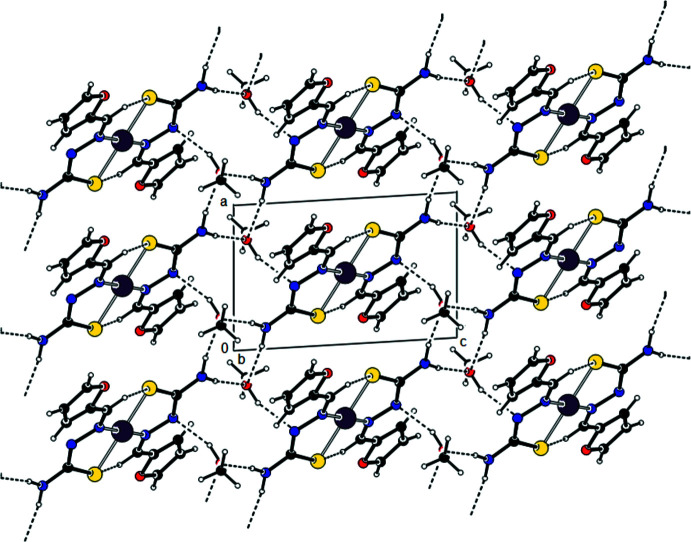
A view along the *b* axis of the crystal packing of the title compound, with hydrogen bonds indicated by dashed lines.

**Figure 4:**
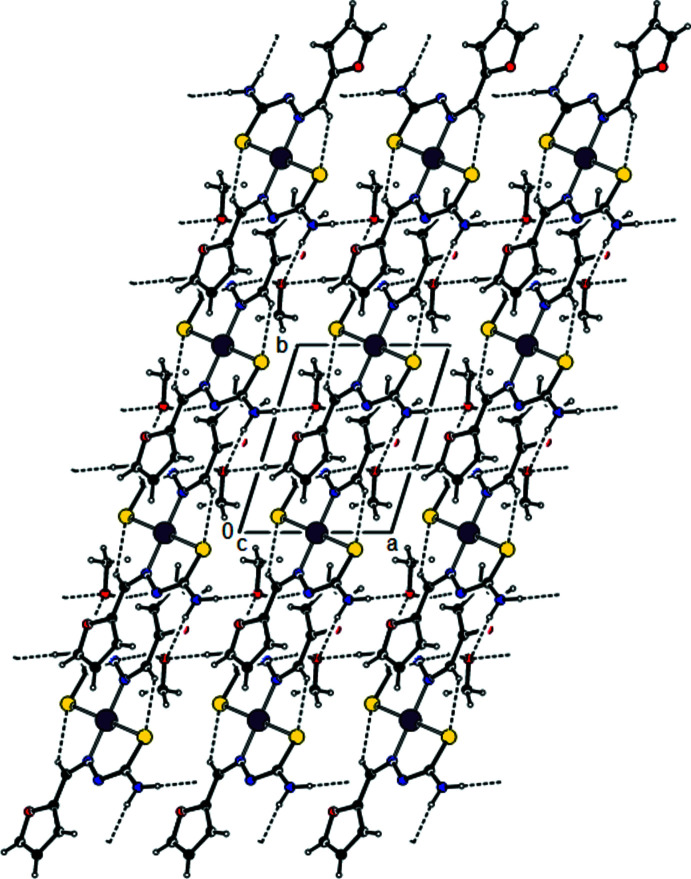
A view along the *c* axis of the crystal packing of the title compound, with hydrogen bonds indicated by dashed lines.

**Table 1:** Hydrogen-bond geometry (Å, °)

*D*—H⋯*A*	*D*—H	H⋯*A*	*D*⋯*A*	*D*—H⋯*A*
O2—H2*O*⋯N2	0.90	1.94	2.788 (3)	156
N3—H3*A*⋯O2^i^	0.90	2.07	2.964 (3)	173
N3—H3*B*⋯O2^ii^	0.90	2.12	3.009 (3)	171
C5—H5⋯S1^iii^	0.93	2.51	3.102 (3)	121

Symmetry codes: (i) 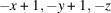 ; (ii) 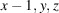 ; (iii) 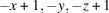 .

**Table 2:** Summary of short inter­atomic contacts (Å) in the title compound

Contact	Distance	Symmetry operation
S1⋯C5	3.55	−1 + *x*, *y*, *z*
H2⋯O1	2.78	2 − *x*, 1 − *y*, 1 − *z*
N2⋯H2*O*	1.94	*x*, *y*, *z*
H3*B*⋯O2	2.12	−1 + *x*, *y*, *z*
H3*A*⋯O2	2.07	1 − *x*, 1 − *y*, 1 − *z*
C1⋯C1	3.51	1 − *x*, 1 − *y*, 1 − *z*
H3*B*⋯H3*A*	2.55	−*x*, 1 − *y*, −*z*
H7*C*⋯H7*C*	2.38	2 − *x*, −*y*, −*z*

**Table 3:** Experimental details

Crystal data	
Chemical formula	[Ni(C_6_H_6_N_3_OS)_2_]·2CH_4_O
*M*_r_	459.19
Crystal system, space group	Triclinic, *P* 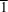
Temperature (K)	296
*a*, *b*, *c* (Å)	6.5394 (11), 8.9611 (15), 10.2020 (15)
α, β, γ (°)	67.965 (5), 79.666 (6), 70.349 (6)
*V* (Å^3^)	520.92 (15)
*Z*	1
Radiation type	Mo *K*α
μ (mm^−1^)	1.16
Crystal size (mm)	0.26 × 0.21 × 0.12

Data collection	
Diffractometer	Bruker APEXII CCD
Absorption correction	Multi-scan (*SADABS*; Bruker, 2008 ▸)
*T*_min_, *T*_max_	0.735, 0.861
No. of measured, independent and observed [*I* > 2σ(*I*)] reflections	8497, 2134, 1633
*R*_int_	0.046
(sin θ/λ)_max_ (Å^−1^)	0.626

Refinement	
*R*[*F* ^2^ > 2σ(*F* ^2^)], *wR*(*F* ^2^), *S*	0.032, 0.088, 1.04
No. of reflections	2134
No. of parameters	125
H-atom treatment	H-atom parameters constrained
Δρ_max_, Δρ_min_ (e Å^−3^)	0.25, −0.21

Computer programs: *APEX4* (Bruker, 2008 ▸), *SAINT* (Bruker, 2008 ▸), *SHELXT2016/6* (Sheldrick, 2015*a* ▸), *SHELXL2016/6* (Sheldrick, 2015*b* ▸, *ORTEP-3 for Windows* (Farrugia, 2012 ▸), *PLATON* (Spek, 2020 ▸).

## supplementary crystallographic information

### Crystal data

**Table:**

[Ni(C_6_H_6_N_3_OS)_2_]·2CH_4_O	*Z* = 1
*M**_r_* = 459.19	*F*(000) = 238
Triclinic, *P*1	*D*_x_ = 1.464 Mg m^−^^3^
*a* = 6.5394 (11) Å	Mo *K*α radiation, λ = 0.71073 Å
*b* = 8.9611 (15) Å	Cell parameters from 2724 reflections
*c* = 10.2020 (15) Å	θ = 2.7–26.4°
α = 67.965 (5)°	µ = 1.16 mm^−^^1^
β = 79.666 (6)°	*T* = 296 K
γ = 70.349 (6)°	Prism, orange
*V* = 520.92 (15) Å^3^	0.26 × 0.21 × 0.12 mm

### Data collection

**Table:**

Bruker APEXII CCD diffractometer	1633 reflections with *I* > 2σ(*I*)
φ and ω scans	*R*_int_ = 0.046
Absorption correction: multi-scan (SADABS; Bruker, 2008)	θ_max_ = 26.4°, θ_min_ = 3.3°
*T*_min_ = 0.735, *T*_max_ = 0.861	*h* = −8→8
8497 measured reflections	*k* = −11→11
2134 independent reflections	*l* = −12→12

### Refinement

**Table:**

Refinement on *F*^2^	Primary atom site location: structure-invariant direct methods
Least-squares matrix: full	Hydrogen site location: mixed
*R*[*F*^2^ > 2σ(*F*^2^)] = 0.032	H-atom parameters constrained
*wR*(*F*^2^) = 0.088	*w* = 1/[σ^2^(*F*_o_^2^) + (0.0459*P*)^2^] where *P* = (*F*_o_^2^ + 2*F*_c_^2^)/3
*S* = 1.03	(Δ/σ)_max_ < 0.001
2134 reflections	Δρ_max_ = 0.25 e Å^−^^3^
125 parameters	Δρ_min_ = −0.21 e Å^−^^3^
0 restraints	

### Special details

**Table:**

Geometry. All esds (except the esd in the dihedral angle between two l.s. planes) are estimated using the full covariance matrix. The cell esds are taken into account individually in the estimation of esds in distances, angles and torsion angles; correlations between esds in cell parameters are only used when they are defined by crystal symmetry. An approximate (isotropic) treatment of cell esds is used for estimating esds involving l.s. planes.

### Fractional atomic coordinates and isotropic or equivalent isotropic displacement parameters (Å^2^)

**Table:**

	*x*	*y*	*z*	*U*_iso_*/*U*_eq_	
Ni1	0.500000	0.000000	0.500000	0.03949 (16)	
S1	0.21552 (9)	0.08780 (8)	0.37725 (6)	0.0544 (2)	
O1	0.8206 (3)	0.4874 (2)	0.41926 (18)	0.0592 (5)	
O2	0.7620 (3)	0.3261 (2)	0.05737 (16)	0.0534 (4)	
H2O	0.634939	0.350050	0.108389	0.080*	
N1	0.5375 (3)	0.2167 (2)	0.39689 (17)	0.0403 (4)	
N2	0.4320 (3)	0.3178 (2)	0.27263 (18)	0.0428 (4)	
N3	0.1595 (3)	0.3562 (2)	0.1429 (2)	0.0558 (6)	
H3A	0.195718	0.448305	0.083360	0.067*	
H3B	0.032648	0.349515	0.126510	0.067*	
C1	0.6747 (4)	0.4539 (3)	0.3592 (2)	0.0446 (5)	
C2	0.8207 (5)	0.6480 (3)	0.3424 (3)	0.0688 (8)	
H2	0.905332	0.703098	0.359617	0.083*	
C3	0.6847 (5)	0.7162 (3)	0.2396 (3)	0.0679 (8)	
H3	0.658018	0.824589	0.173586	0.082*	
C4	0.5872 (4)	0.5926 (3)	0.2494 (3)	0.0544 (6)	
H4	0.483563	0.604491	0.191646	0.065*	
C5	0.6538 (3)	0.2881 (3)	0.4311 (2)	0.0440 (5)	
H5	0.732223	0.223902	0.511529	0.053*	
C6	0.2750 (3)	0.2665 (3)	0.2568 (2)	0.0415 (5)	
C7	0.8122 (6)	0.1616 (4)	0.0563 (3)	0.0883 (10)	
H7A	0.683980	0.143664	0.039468	0.132*	
H7B	0.864640	0.082516	0.146089	0.132*	
H7C	0.922412	0.146101	−0.017494	0.132*	

### Atomic displacement parameters (Å^2^)

**Table:**

	*U*^11^	*U*^22^	*U*^33^	*U*^12^	*U*^13^	*U*^23^
Ni1	0.0337 (2)	0.0371 (3)	0.0388 (2)	−0.01571 (17)	−0.00740 (15)	0.00392 (17)
S1	0.0428 (3)	0.0477 (4)	0.0586 (4)	−0.0248 (3)	−0.0200 (3)	0.0150 (3)
O1	0.0688 (12)	0.0533 (11)	0.0615 (11)	−0.0308 (9)	−0.0191 (8)	−0.0081 (8)
O2	0.0559 (10)	0.0439 (10)	0.0575 (10)	−0.0225 (8)	0.0064 (8)	−0.0116 (7)
N1	0.0346 (9)	0.0423 (10)	0.0352 (9)	−0.0157 (8)	−0.0070 (7)	0.0024 (8)
N2	0.0419 (10)	0.0435 (11)	0.0368 (10)	−0.0213 (8)	−0.0095 (8)	0.0040 (8)
N3	0.0505 (12)	0.0567 (13)	0.0511 (11)	−0.0293 (10)	−0.0223 (9)	0.0121 (10)
C1	0.0482 (13)	0.0444 (13)	0.0435 (12)	−0.0202 (10)	−0.0031 (10)	−0.0114 (11)
C2	0.087 (2)	0.0537 (17)	0.078 (2)	−0.0385 (15)	−0.0121 (16)	−0.0171 (15)
C3	0.095 (2)	0.0426 (15)	0.0671 (18)	−0.0308 (15)	−0.0135 (16)	−0.0068 (13)
C4	0.0675 (16)	0.0402 (14)	0.0540 (15)	−0.0191 (12)	−0.0152 (12)	−0.0063 (12)
C5	0.0457 (12)	0.0435 (13)	0.0366 (12)	−0.0171 (10)	−0.0090 (9)	−0.0005 (10)
C6	0.0350 (11)	0.0408 (12)	0.0391 (12)	−0.0152 (9)	−0.0050 (9)	0.0017 (10)
C7	0.101 (3)	0.0552 (19)	0.111 (3)	−0.0192 (17)	−0.003 (2)	−0.0358 (19)

### Geometric parameters (Å, º)

**Table:**

Ni1—N1	1.9055 (17)	N3—H3A	0.8997
Ni1—N1^i^	1.9055 (17)	N3—H3B	0.9000
Ni1—S1	2.1818 (6)	C1—C4	1.354 (3)
Ni1—S1^i^	2.1818 (6)	C1—C5	1.431 (3)
S1—C6	1.731 (2)	C2—C3	1.323 (4)
O1—C2	1.357 (3)	C2—H2	0.9300
O1—C1	1.384 (3)	C3—C4	1.419 (3)
O2—C7	1.402 (3)	C3—H3	0.9300
O2—H2O	0.9032	C4—H4	0.9300
N1—C5	1.305 (3)	C5—H5	0.9300
N1—N2	1.391 (2)	C7—H7A	0.9600
N2—C6	1.313 (3)	C7—H7B	0.9600
N3—C6	1.332 (3)	C7—H7C	0.9600
			
N1—Ni1—N1^i^	180.0	C3—C2—H2	124.4
N1—Ni1—S1	85.69 (5)	O1—C2—H2	124.4
N1^i^—Ni1—S1	94.31 (5)	C2—C3—C4	107.0 (2)
N1—Ni1—S1^i^	94.31 (5)	C2—C3—H3	126.5
N1^i^—Ni1—S1^i^	85.69 (5)	C4—C3—H3	126.5
S1—Ni1—S1^i^	180.0	C1—C4—C3	106.5 (2)
C6—S1—Ni1	95.83 (7)	C1—C4—H4	126.7
C2—O1—C1	106.13 (18)	C3—C4—H4	126.7
C7—O2—H2O	109.2	N1—C5—C1	127.45 (19)
C5—N1—N2	112.86 (16)	N1—C5—H5	116.3
C5—N1—Ni1	126.69 (14)	C1—C5—H5	116.3
N2—N1—Ni1	120.44 (13)	N2—C6—N3	117.99 (17)
C6—N2—N1	112.74 (15)	N2—C6—S1	122.47 (15)
C6—N3—H3A	116.5	N3—C6—S1	119.54 (16)
C6—N3—H3B	127.8	O2—C7—H7A	109.5
H3A—N3—H3B	114.4	O2—C7—H7B	109.5
C4—C1—O1	109.12 (19)	H7A—C7—H7B	109.5
C4—C1—C5	138.1 (2)	O2—C7—H7C	109.5
O1—C1—C5	112.71 (19)	H7A—C7—H7C	109.5
C3—C2—O1	111.2 (2)	H7B—C7—H7C	109.5
			
C5—N1—N2—C6	−163.93 (19)	N2—N1—C5—C1	2.4 (3)
Ni1—N1—N2—C6	15.0 (2)	Ni1—N1—C5—C1	−176.40 (17)
C2—O1—C1—C4	−0.7 (3)	C4—C1—C5—N1	5.6 (5)
C2—O1—C1—C5	−179.0 (2)	O1—C1—C5—N1	−176.8 (2)
C1—O1—C2—C3	0.3 (3)	N1—N2—C6—N3	178.56 (19)
O1—C2—C3—C4	0.2 (3)	N1—N2—C6—S1	−1.8 (3)
O1—C1—C4—C3	0.8 (3)	Ni1—S1—C6—N2	−8.9 (2)
C5—C1—C4—C3	178.5 (3)	Ni1—S1—C6—N3	170.73 (18)
C2—C3—C4—C1	−0.6 (3)		

Symmetry code: (i) −*x*+1, −*y*, −*z*+1.

### Hydrogen-bond geometry (Å, º)

**Table:**

*D*—H···*A*	*D*—H	H···*A*	*D*···*A*	*D*—H···*A*
O2—H2*O*···N2	0.90	1.94	2.788 (3)	156
N3—H3*A*···O2^ii^	0.90	2.07	2.964 (3)	173
N3—H3*B*···O2^iii^	0.90	2.12	3.009 (3)	171
C4—H4···N2	0.93	2.51	2.882 (3)	104
C5—H5···S1^i^	0.93	2.51	3.102 (3)	121

Symmetry codes: (i) −*x*+1, −*y*, −*z*+1; (ii) −*x*+1, −*y*+1, −*z*; (iii) *x*−1, *y*, *z*.
